# Supplementary material for: The interactome of intact mitochondria by cross-linking mass spectrometry provides evidence for coexisting respiratory supercomplexes
Source: Mol Cell Proteomics. 2017 Dec 8;17(2):216–32. doi: 10.1074/mcp.RA117.000470 (PMC5795388; doi:10.1074/mcp.RA117.000470)
Supplement: Supplemental Data [file supp_RA117.000470_133922_0_supp_23978_fzffwf.zip › spectra_annotation/mito_spectra_annotation/291-1-1-1-3-1.pdf]

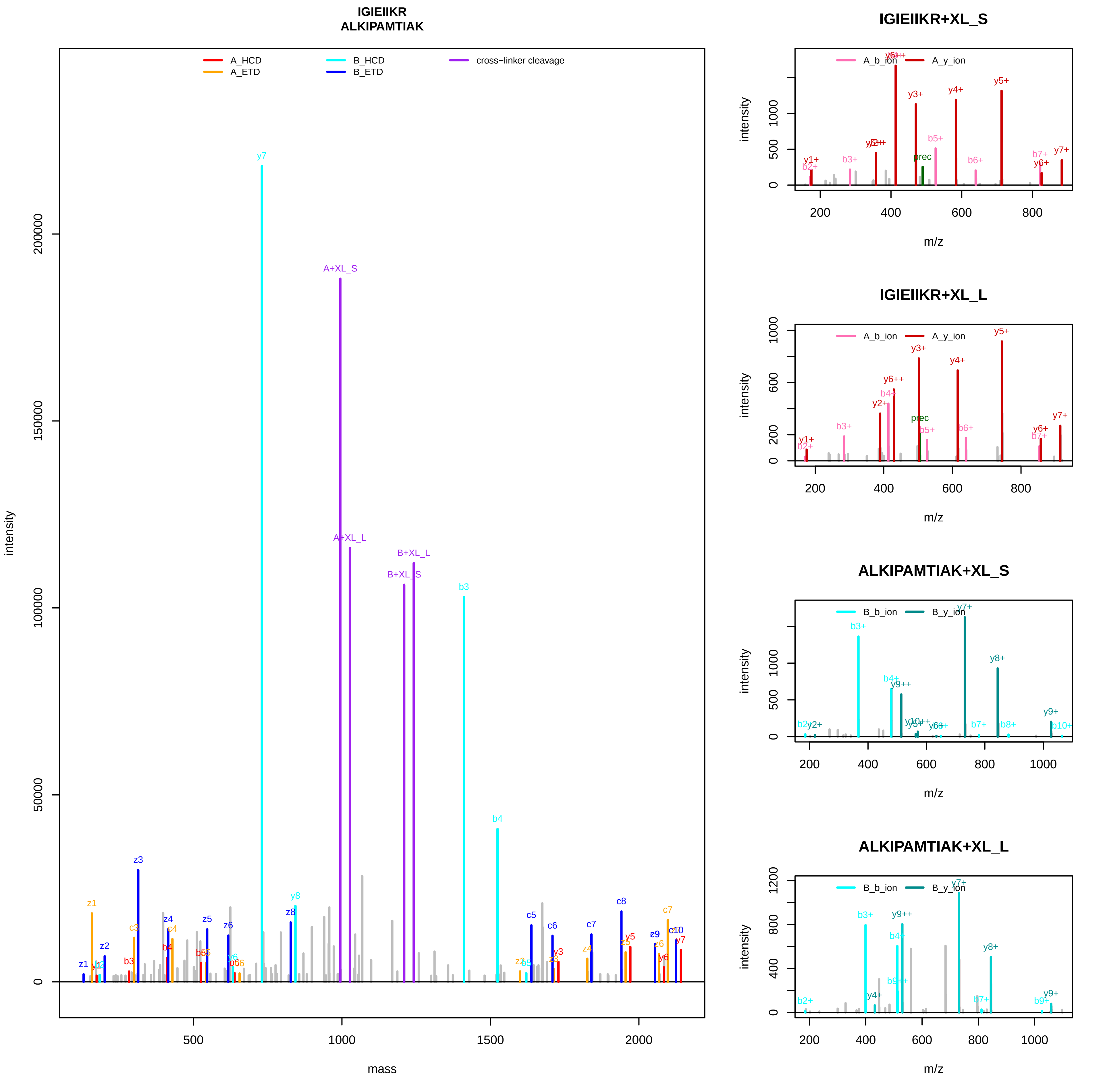
IGIEIIKR
ALKIPAMTIAK
A_HCD
A_ETD
B_HCD
B_ETD
cross−linker cleavage
intensity
mass
0
50000
100000
150000
200000
500
1000
1500
2000
y7
A+XL_S
A+XL_L
B+XL_L
B+XL_S
b3
b4
y8
z8
z1
z2
z3
z4
z5
z6
c3
c4
b3
b4
c5
c6
c7
c8
c9
c10
z9
c7
y5
y6
y7
y3
IGIEIIKR+XL_S
IGIEIIKR+XL_L
ALKIPAMTIAK+XL_S
ALKIPAMTIAK+XL_L
A_b_ion
A_y_ion
B_b_ion
B_y_ion
m/z
prec
y1+
b2+
b3+
y3+
y4+
y5+
b5+
b6+
b7+
y6+
y7+
y2+
y6++
b4+
b2+
b7+
b8+
b9+
b10+
y8+
y9+
y9++
y10++
